# The Opioid Analgesic Reduction Study (OARS)—a comparison of opioid vs. non-opioid combination analgesics for management of post-surgical pain: a double-blind randomized clinical trial

**DOI:** 10.1186/s13063-022-06064-8

**Published:** 2022-02-17

**Authors:** Cecile A. Feldman, Janine Fredericks-Younger, Shou-En Lu, Paul J. Desjardins, Hans Malmstrom, Michael Miloro, Gary Warburton, Brent Ward, Vincent Ziccardi, Daniel Fine

**Affiliations:** 1 School of Dental Medicine, Rutgers University, 110 Bergen Street, Newark, NJ 07103 USA; 2 School of Public Health, Rutgers University, 683 Hoes Lane, Piscataway, NJ 08854 USA; 3 Eastman Institute for Oral Health, University of Rochester, 625 Elmwood Ave, Rochester, NY 14620 USA; 4 College of Dentistry, University of Illinois, 801 S Paulina St, Room 110 (MC 835), Chicago, IL 60612 USA; 5 School of Dentistry, University of Maryland, 650 W Baltimore St, Room 1209, Baltimore, MD 2120 USA; 6 School of Dentistry, University of Michigan, 1515 E. Hospital Drive, Ann Arbor, MI 48109 USA

**Keywords:** Opioids, Acute pain, Opioid-related side effects, Pain management, Combination analgesics, Over-the-counter analgesics, Impact third molar extraction, Satisfaction, Pain interference, Sleep quality

## Abstract

**Background:**

Everyday people die unnecessarily from opioid overdose-related addiction. Dentists are among the leading prescribers of opioid analgesics. Opioid-seeking behaviors have been linked to receipt of initial opioid prescriptions following the common dental procedure of third molar extraction. With each opioid prescription, a patient’s risk for opioid misuse or abuse increases. With an estimated 56 million tablets of 5 mg hydrocodone annually prescribed after third molar extractions in the USA, 3.5 million young adults may be unnecessarily exposed to opioids by dentists who are inadvertently increasing their patient’s risk for addiction.

**Methods:**

A double-blind, stratified randomized, multi-center clinical trial has been designed to evaluate whether a combination of over-the-counter non-opioid-containing analgesics is not inferior to the most prescribed opioid analgesic. The impacted 3rd molar extraction model is being used due to the predictable severity of the post-operative pain and generalizability of results. Within each site/clinic and gender type (male/female), patients are randomized to receive either OPIOID (hydrocodone/acetaminophen 5/300 mg) or NON-OPIOID (ibuprofen/acetaminophen 400/500 mg). Outcome data include pain levels, adverse events, overall patient satisfaction, ability to sleep, and ability to perform daily functions. To develop clinical guidelines and a clinical decision-making tool, pain management, extraction difficulty, and the number of tablets taken are being collected, enabling an experimental decision-making tool to be developed.

**Discussion:**

The proposed methods address the shortcomings of other analgesic studies. Although prior studies have tested short-term effects of single doses of pain medications, patients and their dentists are interested in managing pain for the entire post-operative period, not just the first 12 h. After surgery, patients expect to be able to perform normal daily functions without feeling nauseous or dizzy and they desire a restful sleep at night. Parents of young people are concerned with the risks of opioid use and misuse, related either to treatments received or to subsequent use of leftover pills. Upon successful completion of this clinical trial, dentists, patients, and their families will be better able to make informed decisions regarding post-operative pain management.

**Trial registration:**

ClinicalTrials.govNCT04452344. Registered on June 20, 2020

## Administrative information

Note: the numbers in curly brackets in this protocol refer to Standard Protocol Items: Recommendations for Interventional Trials (SPIRIT) checklist item numbers. The order of the items has been modified to group similar items (see http://www.equator-network.org/reporting-guidelines/spirit-2013-statement-defining-standard-protocol-items-for-clinical-trials/).

**Table:** 

Title {1}	The Opioid Analgesic Reduction Study (OARS): A comparison of opioid vs. non-opioid combination analgesics for management of post-surgical pain study protocol for a double-blind randomized multi-center clinical trial.
Trial registration {2a and 2b}.	ClinicalTrials.gov NCT04452344. Registered on June 20, 2020
Protocol version {3}	Version 5.0; July 30, 2021
Funding {4}	Supported by National Institute of Dental and Craniofacial of the National Institutes of Health awards under award numbers UG3DE028860 and UH3DE028860.
Author details {5a}	**Primary and Corresponding Author** Cecile A. Feldman, DMD, MBA Dean and Professor Rutgers University – School of Dental Medicine and Professor Rutgers University – School of Public Health 110 Bergen Street Newark, NJ. 07103 Phone: 973-972-4634 e-mail: feldman@rutgers.edu **Additional Contributors to the Protocol** Janine Fredericks-Younger, DMD Rutgers University, School of Dental Medicine 110 Bergen Street, Room D813 Newark, NJ 07101 Phone: (973) 972-1679 frederja@sdm.rutgers.edu Paul J. Desjardins, DMD, Ph.D. Rutgers University, School of Dental Medicine 110 Bergen Street, Room B815 Newark, NJ 07101 Phone: (973) 762-4430 paul.j.desjardins@gmail.com Shou-En Lu, PhD Rutgers University, School of Public Health 683 Hoes Lane Piscataway, NJ 08854 Phone: (732) 235-5764 sl1020@sph.rutgers.edu Hans Malmstrom, DDS University of Rochester, Eastman Institute for Oral Health 625 Elmwood Ave. Rochester, NY 14620 Phone: (585) 273-5087 hans_malmstrom@urmc.rochester.edu Michael Miloro, DMD, MD University of Illinois, College of Dentistry 801 S Paulina St, Room 110 (MC 835) Chicago, IL 60612 Phone: (312) 996-1052 mmiloro@uic.edu Gary Warburton, MD, DDS University of Maryland, School of Dentistry 650 W Baltimore St, Room 1209 Baltimore, MD 2120 Phone: (410) 706-7060 gwarburton@umaryland.edu Brent Ward, DDS, MD University of Michigan, School of Dentistry 1515 E. Hospital Drive Ann Arbor, MI 48109 Phone: (734) 936-5950 bward@med.umich.edu Vincent Ziccardi, DDS, MD Rutgers University, School of Dental Medicine 110 Bergen St., Room B854 Newark, NJ 07101 Phone: (973) 972-7462 ziccarvb@sdm.rutgers.edu Daniel Fine, DMD Rutgers University, School of Dental Medicine 110 Bergen St., Room B854 Newark, NJ 07101 Phone: (973) 972-3728 finedh@sdm.rutgers.edu
Name and contact information for the trial sponsor {5b}	Rutgers, The State University of New Jersey, Rutgers School of Dental Medicine, 110 Bergen Street, Newark, New Jersey, USA
Role of sponsor {5c}	The content is solely the responsibility of the authors and does not necessarily represent the official views of the National Institutes of Health.

## Introduction

The goal of this study is to provide health care professionals, including dentists, with the best possible evidence for clinical decision-making when selecting analgesics for acute post-surgical pain management. A double-blind, stratified randomized clinical trial is being conducted to test the hypothesis that a combination of over-the-counter non-opioid-containing analgesics is at least as, if not more, effective (non-inferior) than the most commonly prescribed opioid analgesic. The impacted third molar extraction model is used due to the predictable severity of post-operative pain and generalizability of results, as well as the fact that dentists prescribe approximately one-third of all opioid prescriptions for adolescents in the USA.

## Background and rationale {6a}

### Description of health problem

Opioid-related deaths are rising alarmingly in the USA [1]. Opioid overdose deaths have more than quadrupled since 1999 [2] accounting for over 49,860 deaths in 2019 [3]. Unprecedented increases in opioid overdose deaths have occurred during the COVID-19 pandemic with a 57.7% increase between May 2019 and May 2020 [4]. Between 1999 and 2019, nearly 247,000 Americans have died due to overdoses involving prescription opioids [5]. Many patients do not properly secure or dispose of their unused opioid medications, leaving them accessible to others who may initiate or feed an addiction [6, 7].

Millions of tablets of 5 mg hydrocodone-containing combinations are prescribed after third molar extractions each year in the USA [8]. High school students who receive an opioid prescription are 33% more likely than those who do not receive an opioid prescription to eventually misuse opioids [9] contributing to an upsurge in deaths among 18- to 25-year-olds [10].

Dentists are among the leading prescribers of opioid analgesics [11], accounting for 12% of all prescriptions for immediate-release opioids [12]. They rank fourth among medical specialties for their opioid-prescribing rates, writing 18.5 million opioid prescriptions per year [13]. Over 76% of these opioid prescriptions have been for hydrocodone combination products. Adolescents and young adults receive more than 11% of dentist-prescribed opioids [14]. This finding of opioid-prescribing prevalence for adolescents is consistent with other studies and the assessment of acute opioid prescriptions for youth [11, 15]. Dentists write about 31% of opioid prescriptions for young patients aged 10 to 19 [8] with about 61% of 14- to 17-year-olds receiving opioid prescriptions following third molar extractions [16]. Opioid-seeking behaviors have been linked to the receipt of initial opioid prescriptions following third molar extraction, a procedure that 3.5 million young adults undergo annually.

A trial has been designed to minimize unnecessary opioid prescribing by comparing the effectiveness of a combination of non-opioid-containing analgesics (ibuprofen and acetaminophen) to the most commonly prescribed combination opioid-containing analgesic (hydrocodone with acetaminophen). This study builds upon previous studies and data, and addresses prior study limitations, and reinforces the generalizability of this information for health care professionals.

### Previous study weaknesses

Ibuprofen/acetaminophen and opioid/acetaminophen analgesic combinations are commonly prescribed after dental surgery [17–29]. Both medication options have been shown to be more effective than placebo and have been tested extensively as evidenced by several prior systematic reviews [17, 19, 27–36]. Completed studies, however, are limited for the following reasons: they only compared the specific analgesic drug against a placebo; they frequently utilize a single dose which may miss the peak post-surgical pain period [37]; they wait for the onset of pain before administering the analgesic drug; they do not consider sex differences [37–41]; they do not examine heterogeneity issues; they have small sample sizes and do not test for non-inferiority [42, 43]; they use dosages that are either not commercially available, or enable patients to easily exceed the recommended US Federal Drug Administration (FDA) maximum dosage; and/or they follow patients for just several hours after surgery [34]. Furthermore, those earlier studies looked primarily at pain outcomes, prohibited other commonly prescribed treatments like corticosteroids, and limited the extractions to either 2 molars or 4 molars. Therefore, there are an array of studies with limited relevance to typical clinical decision-making, with varying analgesic medication dosages [37], and with varying lengths of follow-up periods; these factors make decisions by both health care providers and patients difficult and subject to individual preference and bias.

### Importance of the study

With each opioid prescription, the risk for opioid misuse or abuse by the patient or others who may have access to the medications increases. Recent studies have shown that exposing young adults to an opioid analgesic increases their risk of future opioid use, with as much as 37% of non-medical opioid use by high school seniors originating from leftover opioid prescriptions in their or their friend’s or families’ households [44]. If an alternative analgesic is found to be non-inferior to the most commonly prescribed opioid for impacted third molar extractions, the overall number of opioid prescriptions would be reduced. In turn, with decreased opioid substrate remaining in the community, the number of young adults at risk for opioid abuse and addiction via drug diversion has the potential to be significantly reduced [45, 46].

This trial addresses numerous limitations of previous studies and will provide health care professionals, including dentists, with the best possible evidence for clinical-based decision-making when selecting upon analgesics for acute post-surgical pain management of third molars with generalizability to other similar pain evoking procedures. This study uses the impacted 3rd molar extraction model due to the predictable severity of the post-operative pain and generalizability of results [47–51]. The third molar extraction model has been widely used to assess interventions to treat acute post-surgical pain due to its reproducibility and its sensitivity.

## Objectives {7}

### Aim 1—Pain management and patient satisfaction

We hypothesize that a combination of acetaminophen and ibuprofen (NON-OPIOID) is non-inferior to the most commonly prescribed opioid analgesic, hydrocodone with acetaminophen (OPIOID), with respect to pain management (hypothesis 1a) and NON-OPIOID patient satisfaction is better than OPIOID patient satisfaction (hypothesis 1b). These hypotheses are being tested via individual patient reporting of their post-operative pain experience in a daily electronic diary (eDiary) and rating their overall satisfaction using a software application developed for electronic phones and tablets.

### Aim 2—Adverse events, daily function, and opioid-seeking behavior

We hypothesize that patients receiving NON-OPIOID will experience fewer and less severe adverse events, experience superior sleep and daily function, and exhibit less opioid-seeking behavior than patients receiving OPIOID (hypothesis 2a). In addition, we hypothesize that patients receiving 5 days of opioid-containing analgesics will have tablets/capsules remaining after their acute pain episode has been resolved (hypothesis 2b). Specifically, the following will be examined:

- *Adverse effects*: (i.e., constipation, diarrhea, dizziness/light headedness, euphoria, headache, nausea, tired/sleepy, vomiting, itching, and urinary retention) by patient reports in their electronic diaries and emergency call/visit logs
- *Daily function****:*** ability to carry out their normal activities (determined by the mean normal-daily-function rating) by patient rating in their daily diary
- *Sleep quality:* sleep quality either from data obtained by an electronic sleep monitor device or by patient rating in their daily diary
- *Future opioid-seeking behavior:* number of NON-OPIOID patients filling an opioid prescription within 6 months following the study post-operative period by querying Prescription Database Monitoring Program databases
- *Diversion:* number of doses remaining return bottles at the time of the post-operative visit or by electronic medical bottle monitoring, which is normally well past the time period in which patients require analgesics.

### Aim 3—Clinical protocol/decision support tool

A model that recommends which analgesic to prescribe along with the number of tablets to maximize a patient’s overall satisfaction will be developed. Independent variables going into the model include a patient’s pain sensitivity, expectations, number and location of extractions (maxillary vs. mandibular), level of surgical difficulty, surgical time, education level, gender, race and ethnicity, and age.

## Trial design {8}

A multi-site, double-blind, prospective, stratified, randomized clinical trial has been designed and is being conducted. Pragmatic trials are designed to measure effectiveness based upon real-world clinical practice and patient variability. The study design therefore allows for multiple 3rd molar extractions and, after the first study analgesic dose, an “as needed for pain” dosing schedule.

The planned dosages were selected because both study product dosages can be safely increased should adequate pain relief not be obtained. Participants are stratified based upon gender due to differences in prescribing patterns and pain tolerance [38–41] and are randomized at a 1:1 ratio. Each arm enrolls 450 participants per gender for a total enrollment of 1800 participants. The study is designed to evaluate the non-inferiority of *NON-OPIOID* in real-life clinical practice conditions, providing results that can be generalized and applied to routine practice settings described below.

**Fig. 1:**
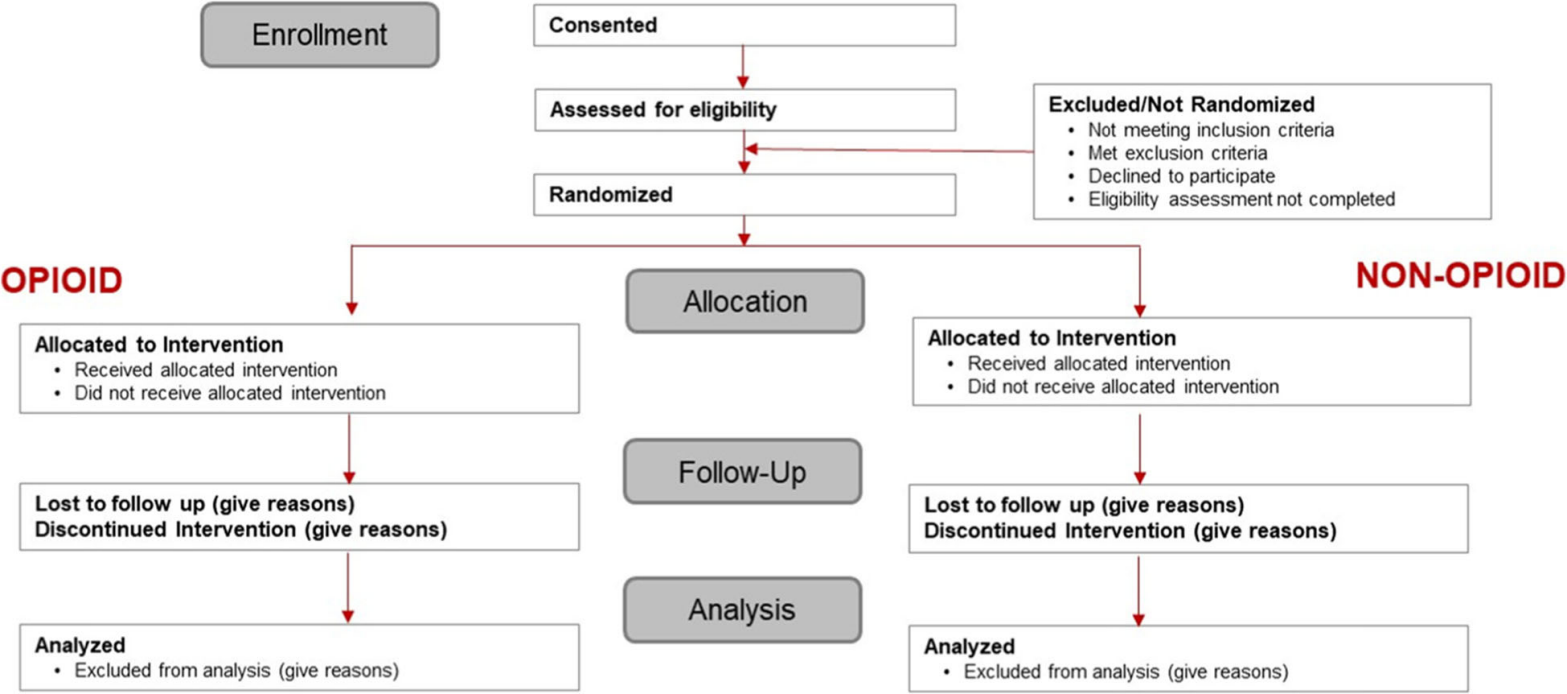
Flow diagram

Participants are asked to complete several questionnaires, to take a pain medication provided, and to wear an activity monitor for 72 h. Participants complete questionnaires during the surgery visit and post-operative visit. During the post-operative period, participants receive electronic messages, via text or e-mail, in the morning and evening reminding them to complete an eDiary entry on their smartphone to record pain levels, pain interference, sleep quality, and medication usage. Figure 1 illustrates the flow of the trial.

## Methods: participants, interventions, and outcomes

### Study setting {9}

*Target sample size:* 900 female, 900 male, 1800 total

*Target sample size by gender, race, ethnicity, and age:* The study population is drawn from outpatient adult patients seeking extraction of impacted mandibular 3rd molars in the following communities: Ann Arbor, MI; Baltimore, MD; Chicago, IL; Newark, NJ; and Rochester, NY. Sites selected are all located in diverse communities and serve a diverse patient population. It is anticipated that at least 32% will be African American and 17% will be Hispanic or Latino. While most participants seeking third molar extraction are young adults, adults of any age (> 18) can be enrolled.

A stratified random sample design with stratification on gender is used to ensure that one-half of enrolled participants are female and one-half are male.

### Eligibility criteria {10}

The study population is comprised of individuals, 18 years and older, requiring extraction of one or more partially impacted or fully impacted mandibular 3rd molars.

#### Inclusion criteria

An individual must meet all of the following criteria to be eligible to participate in the study: understand the informed consent; provide a signed and dated informed consent form; understand all directions for data gathering instruments in English; be willing and able to comply with all study procedures, including having a smart phone, and be available for the duration of the study; plan to undergo extraction of one or more partially or fully impacted mandibular 3rd molars; be 18 years or older; be in good general health as evidenced by medical history; and, if female gender, must agree to use contraceptive pill, intra-uterine device, condoms, or abstinence while participating in the study.

#### Exclusion criteria

Participants who self-report the following history are excluded from study participation: history of gastrointestinal bleeding and/or peptic ulcer; renal disease (excluding kidney stones); hepatic disease; cardiovascular disease (myocardial infarction or stroke within the past 6 months); bleeding disorder; respiratory depression; prior respiratory effect of an opioid or other anesthetic drugs that required respiratory support post-operatively; active or untreated asthma; allergic reaction to ibuprofen, acetaminophen, hydrocodone, and/or anesthesia, consumption of 3 or more alcoholic drinks each day and/or has a history of alcoholism; history of drug or alcohol abuse (excludes marijuana use); family history of drug or alcohol abuse in a first-degree relative; has had no more than one opioid prescription filled within the past 12 months; and currently pregnant or lactating. In addition, individuals currently taking any of the following medications: CYP3A4 inhibitor, such as macrolide antibiotics (e.g., erythromycin), azole-antifungal agents (e.g., ketoconazole), and protease inhibitors (e.g., ritonavir), which may increase plasma concentrations of hydrocodone bitartrate and acetaminophen and prolong opioid adverse reactions, and which may cause potentially fatal respiratory depression or CNS depressants (including benzodiazepines), are excluded.

Participants are free to withdraw from participation in the study at any time upon request.

An investigator may discontinue an individual’s participation in an intervention or withdraw an individual from the study if Exparel (bupivacaine liposome injectable suspension) is used during the extraction procedure; the participant has a serious adverse event requiring hospitalization; any clinical adverse event (AE), laboratory abnormality, or other medical condition or situation occurs such that continued participation in the study would not be in the best interest of the participant; or the participant meets an exclusion criterion (either newly developed or not previously recognized) that precludes further study participation.

### Who will perform the informed consent procedure? {26a}

Consent is obtained by research coordinators at each clinical site. They are trained in human subject protection, Good Clinical Practice, and the OARS study protocol and procedures. The coordinator explains the research study to the potential participant undergoing mandibular 3rd molar extraction and answer any questions that may arise. Extensive discussion of risks and possible benefits of study participation is provided. A consent form describing in detail the study procedures and risks is given to the participant. Consent forms and recruitment materials are Institutional Review Board approved. Each participant signs the informed consent document prior to any study-related assessments or procedures. The participant may withdraw consent at any time throughout the course of the study. A copy of the executed consent form (either paper or electronic) is provided to the patient at the time of consent for their records.

### Additional consent provisions for collection and use of participant data and biological specimens {26b}

The consent form notifies participants that de-identified information may be used or distributed to other investigators without obtaining additional informed consent. A subject who has withdrawn can also withdraw consent for use of data collected about themselves by sending a request to the principal investigator in writing. This trial does not involve collecting biological specimens for storage.

## Interventions

### Explanation for the choice of comparators {6b}

Two orally administered analgesics are being compared: (1) hydrocodone 5 mg/acetaminophen 300 mg and (2) ibuprofen 400 mg/acetaminophen 500 mg over a post-operative period.

*OPIOID:* This trial uses hydrocodone 5 mg plus acetaminophen 300 mg [52–58]. Hydrocodone represents over 60% of opioid prescriptions for dentistry with codeine being the second most commonly prescribed opioid [14, 43, 59–64]. Codeine with a morphine milligram equivalent (MME) dose of .15 is less effective than hydrocodone, which has an MME of 1 (i.e., hydrocodone is significantly more powerful than codeine) [65].

*NON-OPIOID:* The NON-OPIOID protocol calls for acetaminophen 500 mg (simulating 1 extra strength Tylenol tablet) and ibuprofen 400 mg (simulating 2 Advil or Motrin tablets) which provides anti-inflammatory effects [30–32, 39, 65–73]. Clinical studies have shown that acetaminophen combined with ibuprofen is more effective than either alone in managing acute post-operative pain [29, 39, 65, 74].

Twenty doses are dispensed to each subject. All treatments have an equal appearance.

### Intervention description {11a}

Participants are directed to take 1 dose of either OPIOID or NON-OPIOID immediately after surgery and then 1 dose every 4–6 h as needed for pain. If a dosage is not effective within an hour, a participant can take an additional dose, up to 6 total doses per day. In the event pain management remains insufficient, the subject is instructed to call the on-call surgeon. The on-call surgeon assesses the situation and may instruct the participant to take up to 2 additional doses or determine whether rescue medication (oxycodone 5 mg) or an emergency visit is required.

### Criteria for discontinuing or modifying allocated interventions {11b}

Subjects who require rescue medication due to insufficient pain management with study analgesic are prescribed oxycodone 5 mg in addition to the study analgesic. In addition, subjects who have adverse reactions which cannot be tolerated by the subject and/or subjects who have severe adverse reactions related to study analgesics are instructed to discontinue the use of study analgesics.

### Strategies to improve adherence to interventions {11c}

During the informed consent process, a thorough discussion with potential participants ensures that participants understand trial expectations. Subjects are provided written instructions after surgery. Short message service (SMS) texts are sent each morning and evening during the post-operative period. Subjects receive SMS texts during the post-operative period between surgery and the post-operative visit in the morning and evening. In addition, research coordinators call participants to answer any questions and remind them of the study protocol.

### Relevant concomitant care permitted or prohibited during the trial {11d}

Long-lasting local anesthetics such as Exparel and Marcaine (bupivacaine) are not permitted to be used.

### Provisions for post-trial care {30}

Six months after completion of the trial, subjects who obtain additional opioids are offered, free-of-charge, an opioid addiction screening counseling session.

### Outcomes {12}

Table 1 summarizes the outcome measures. The primary outcome measures for this study are patient-perceived pain [75] levels observed for 7 days following 3rd molar extraction and overall satisfaction [76] with the management of acute post-surgical pain. Secondary outcome measures include (1) frequency and magnitude of adverse events [76], (2) ability to sleep [77] and pain interference [78] with normal daily activities, (3) potential diversion defined as tablets remaining in households and returned at the follow-up visit, and (4) number of opioid prescriptions within 6 months after study analgesic use.

**Table 1:** Outcome measures

Brief description/justification of outcome measure	Outcome measured by	Timing
***Primary outcome measures***		
***Pain****:* For pain level, Brief Pain Inventory (BPI) using the Numerical Rating Scale (NRS) was chosen because: • BPI with NRS is widely used and accepted • Reliability and validity for BPI and NRS have been established • BPI with NRS is clinically relevant (patients want to minimize pain experienced after surgery) • BPI with NRS is a sensitive measure • BPI with NRS allows for direct comparisons across studies ***Satisfaction****:* For satisfaction, overall satisfaction questions from the Pain Treatment Satisfaction Scale (PTSS) were chosen because: • While patients want to minimize pain, patients are willing to tradeoff some pain relief to minimize side effects, maintain their ability to sleep, maintain their ability to engage in normal activities, and minimize exposure to opioids • PTSS has been shown to be valid and reliable	***Pain****:* NRS is used on a scale of 0 to 10 where 0 = no pain and 10 = worst imaginable pain for average pain level, pain at its worst, pain at its least, pain experiencing now. ***Satisfaction*** (measures from PTSS): • How satisfied are you with the *time* that it takes your pain medication to work? (scale: 1 = very satisfied, 2 = satisfied, 3 = neither satisfied nor dissatisfied, 4 = dissatisfied, 5 = very dissatisfied) • How satisfied are you with the *level of amount* of pain relief provided by your pain medication? (scale: 1 = very satisfied, 2 = satisfied, 3 = neither satisfied nor dissatisfied, 4 = dissatisfied, 5 = very dissatisfied) • How satisfied are you with the *duration* of pain relief provided by your pain medication? (scale: 1 = very satisfied, 2 = satisfied, 3 = neither satisfied nor dissatisfied, 4 = dissatisfied, 5 = very dissatisfied) • *Overall*, how satisfied are you with your pain medication? (scale: 1 = very satisfied, 2 = satisfied, 3 = neither satisfied nor dissatisfied, 4 = dissatisfied, 5 = very dissatisfied) • Overall, how does your level of pain relief meet your expectations of pain relief? (scale: 1 = greatly exceeds my expectations, 2 = somewhat exceeds my expectations, 3 = meets my expectations, 4 = does not quite meet my expectations, 5 = does not meet my expectations at all) • Do you think that your pain medication could be more effective in relieving your pain? (scale: 1 = yes, definitely, 2 = probably yes, 3 = I do not know, 4 = probably not, 5 = definitely not) Participants report their pain experience and rate their overall satisfaction using a REDCap application developed for electronic phones and tablets.	***Pain****:* • Visit 1 (in the last 24 h) • Each morning days 2 to 8 • Each evening days 1 to 7 • Visit 2 (in the last 24 h) ***Satisfaction****:* Satisfaction recorded during post-op visit (visit 2)
***Secondary outcome measures***		
***Adverse events****:* As medications have side effects, a list of possible adverse events (side effects) related to the intervention has been developed, and participants are asked if they are experiencing any of them. (This is separate from serious adverse events which are captured. Serious adverse events result in a participant being exited from the study. An analysis of serious adverse events is included in the study analysis.) ***Sleep quality****:* • Pain and Sleep Questionnaire 3-item index (PSQ-3) was selected because it is a validated measure and because of its ease of use for the eDiary on a smart phone. • A question from the PTSS was selected because it provides an overall rating of the quality of sleep. ***Pain interference (daily function)****:* The PROMIS Short 6b was selected because it is a standard NIH measure of pain interference and can be recorded during the post-operative visit. ***Future opioid-seeking behavior****:* The PDMP is accessed; 6 months was selected because it is the maximum follow-up time which could be completed within the study time frame. ***Potential diversion****:* Participants are instructed to bring the pill bottle and unused capsules to the follow-up appointment.	***Adverse events****:* • Adverse events include excessive fatigue or drowsiness, inability to concentrate, nausea, diarrhea, dizziness, constipation, skin rashes, stomach aches, heartburn, vomiting, euphoria, headache, urinary retention, and unintentional weight gain with a binary yes/no scale. Self-reported binary response (yes/no) is ascertained. If yes, was the adverse event bothersome to a minor or major extent? o For visit 1, participants are asked how much were you bothered by … over the last 24 h. o At the time of getting up in the morning, participants are asked how much were you bothered with …. during the night o Right before going to sleep at night, participants are asked how much they were bothered by … during the day. o For visit 2, participants are asked how much were you bothered by … over the last 24 h. ***Sleep ability****:* • From the PSQ-3: (a) Last night did you have trouble falling asleep? (b) Last night were you awakened by pain during the night? (c) Were you awakened by pain this morning? {binary yes/no scale during the post-operative period; NRS scale where 0 = never, 10 = always at visits 1 and 2} • From PTSS: rating the overall quality of last night’s sleep {NRS where 0 = excellent and10 = very poor} • From ActiGraph: Sleep quality is monitored, and data collected ***Pain interference****:* • PROMIS Short 6b: During the post-operative period, how much did pain interfere with your day to day activities, work around the home, ability to participate in social activities, enjoyment of life, the things you usually do for fun, enjoyment of social activities, household chores, family life, your ability to concentrate, enjoyment of recreational activities, tasks away from home {scale: 1 = not at all, 2 = a little bit, 3 = somewhat, 4 = quite a bit, 5 = very much} and how often did pain keep you from socializing with others? {scale: 1 = never, 2 = rarely, 3 = sometimes, 4 = often, 5 = always} ***Future opioid-seeking behavior****:* • # of new opioid prescriptions recorded in the Prescription Monitoring Database Program at approximately 6 months following the surgical procedure ***Potential diversion****:* • # returned capsules at visit 2 determined by counting the returned capsules or via electronic monitoring device • # unaccounted for capsules at visit 2 (not recorded as used and not returned) Participants report whether they have experienced each adverse event and rate their sleep ability and pain interference in a daily electronic diary using a REDCap application developed for electronic phones and tablets.	***Adverse events****:* • Visit 1 • Each night for days 2–8 • Each day for days 1–7 • Visit 2 ***Ability to sleep****:* • Visit 1 • Each morning on days 2 thru 8 and • Visit 2 (in the last 24 h) ***Pain interference****:* • Visit 1 • Each evening on days 1 through 7 • Visit 2 ***Future drug-seeking behavior****:* • Opioid prescriptions filled within 6 months after visit 2 ***Potential diversion****:* • Visit 2

### Participant timeline {13}

#### Expected duration of subject participation

**Fig. 2:**
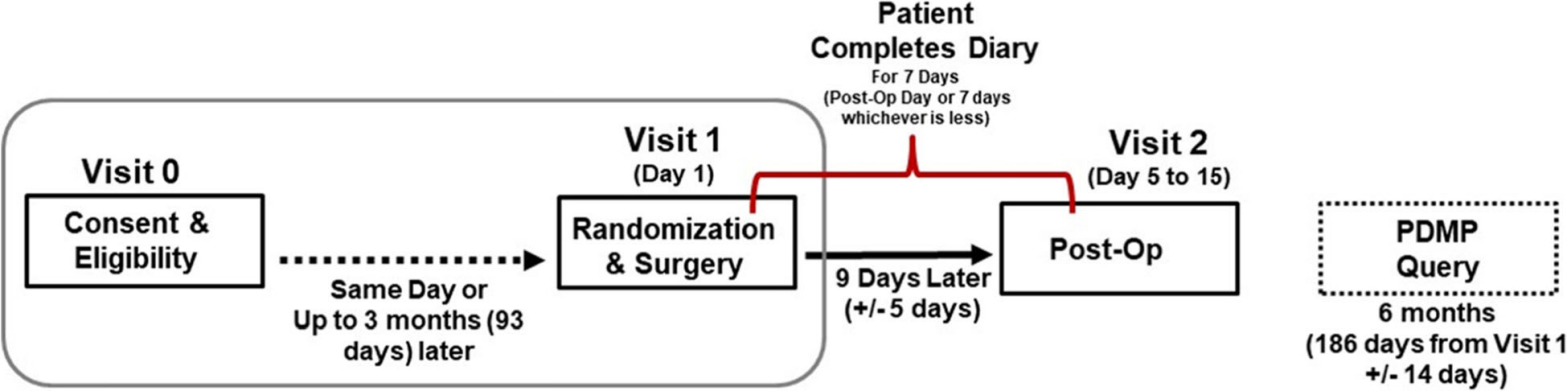
Participant timeline

Subjects participate in the study for the time between their impacted 3rd molar extraction procedure until their post-operative visit which normally occurs approximately 1 week later (9 days ±5 days). Following state laws and regulations, the PDMP is retrospectively queried 6 months post-surgery to ascertain information about any opioid-containing prescriptions that may have been filled during this post-surgery time period. Figure 2 shows the participant timeline.

#### Sequence of procedures and duration of study period

Table 2 provides the schedule of events.

**Table 2:** Schedule of events

			Post-Operative Period						
Procedures	Screening (Visit 0)	Study Visit 1: Surgery (Day 1)	Upon Waking in the Morning (Days 2 to 10 +/-5)	When taking Pain Meds (Days 1 to 10 +/-5)	Before Going to Sleep in the Evening (Days 1 to 9 +/-5)	Intermediate Visit / Hospitalization or Fatality Days 1 to 10 (+/- 5)	Post Operative Visit Study Visit 2 (Day 10 +/-5)	Withdrawal or Termination	PDMP Query (Visit 2 plus 186 days +/- 14 days)
Signed Consent Form	X								
Assessment of Eligibility Criteria (including review of medical history and concomitant medications)	X	X							
Study Intervention		X	X	X	X				
Pain Assessment		X							
Pain Interference Assessment					X		X		
Sleeping Quality			X				X		
Assessment of Adverse Events			X		X		X	X	
Adverse Events and Serious Adverse Event Reporting						X			
Obtain Satisfaction							X		
Determination of Tablets for Diversion							X		
Premature Exit study Documentation								X	
PDMP Inquiry									X

### Sample size {14}

To address the possible heterogeneity in pain management and side events in men and women due to sex differences and differences in pain tolerance, a subgroup analysis in men and women will be performed separately to ensure the hypothesized differences between the analgesic groups remain.

#### Sample size and power

To test if the non-opioid analgesics are non-inferior to opioid analgesics for pain management (*hypothesis 1a*), we chose a small and clinically non-significant difference *d* = 1.0 as the non-inferiority margin and assumed the maximum standard deviation (SD) of the daily pain intensity to be 3.6, based on data from prior studies [42, 79, 80]. With 90% power and alpha = 0.625% (one-sided, after Bonferroni correction for 4 tests of pain experience for day of surgery, day 1 and day 2 post-surgery, and last day of follow-up) [81], we need at least *370* participants per group to test non-inferiority of non-opioid analgesics. To account for 15–20% loss of follow-up and missing data, and other factors not included in the sample size estimation, we will recruit *1800 participants* with *450 participants* × 2 analgesic groups × 2 gender subgroups (men and women). *Power for subgroup analysis*: With *n* = 370/group (after attrition/missing data, etc.), non-inferiority margin *d* = 1.0, and alpha = 0.3125% (one-sided), we have 85% power to test non-inferiority of non-opioid analgesics in men and women separately.

To compare patient satisfaction (*hypothesis 1b*), we estimated the minimal detectable difference in proportions of positive rating between NON-OPIOID vs. OPIOID for *n* = 740/analgesic group (entire sample) and *n* = 370/analgesic group (subgroup) analyses, after attrition/missing data. Assuming the proportion of positive rating (extremely satisfied and satisfied) for NON-OPIOID in our study is about 82%, similar to data in Daniels et al. [43], our study has 90% power to test a minimal difference of 7% (82% vs. 75%) for *n* = 740/analgesic group (entire sample, 2-sided alpha = 5%) and 11% (82% vs. 71%) for *n* = 370/analgesic group (subgroup analysis, 2-sided alpha = 2.5%), in comparing the proportion of positive rating of patient satisfaction. For continuous measures (e.g., mean normal-daily-function ratings and the sleep quality scores (*aim 2*)), our study has 90% power to test a small effect size, Cohen’s *d* of 0.20, for the entire sample (2-sided alpha = 1.25%, after Bonferroni corrections for 4 tests for day of surgery, day 1 and day 2 post-surgery, and last day of follow-up) and Cohen’s *d* = 0.30 for the subgroup analysis (2-sided alpha = 0.625%).

#### Consideration of within-site correlation

In the sample size and minimal effect size estimations, we tentatively assumed all individuals are independent. However, in our study, the randomization will be performed *within each site.* When the correlation between responses among participants within the same site is positive, randomizing participants within site can increase power: Consider a site of 2 m participants and each patient is randomized to one of two treatments at a 1:1 ratio. Assume that the within-site correlation is *r* > 0 and denote the standard deviation of each individual’s response by *σ*. The variance of the mean difference in the responses between two treatments is 2*σ*^2^/m*(1-*r*). It is smaller than 2*σ*^2^/*m*, the variance of the same difference when individuals are mutually independent. The smaller variance implies greater efficiency/power. Therefore, we expect our estimated sample size and minimal effect size to be more conservative than they should have been.

### Recruitment {15}

#### Recruitment strategies

Each clinical site recruits study participants as they report to the oral and maxillofacial surgery or general dentistry clinic for 3rd molar extraction consultation or surgery appointments. Each site has study staff who review the clinic schedule at least each week for potential study candidates. When patients report to the clinic for a 3rd molar extraction consult or procedure, study staff ascertain interest in study participation if at least one partial or full bony mandibular 3rd molar extraction is planned. Sites advertise for the study by word of mouth and/or advertising through flyers, social media, e-mail, and/or ads in local/college newspapers.

## Assignment of interventions: allocation

### Sequence generation {16a}

Participants were randomized to either the opioid or non-opioid analgesic group at the 1:1 ratio, stratified by gender at each site. Site-specific pre-determined random number sequences, with a block of 4 containing 2 opioid and 2 non-opioid assignments in a random order, were generated by staff at Data Management Statistical Analysis Core (DMSA) using R software. The randomization code was generated, and labels are created during the preparation phase when the treatment packets are prepared so that each packet is prepared and labeled with the packet identification number according to the randomization sequence. Complete randomization code for each site is stored in REDCap and only the DMSA and Clinical Protocol Coordinating Core (CPCC) staff have access to it.

### Concealment mechanism {16b}

Existing FDA-approved caplets are placed into opaque capsules and then filled with powder fill. Study analgesics are provided in 2 different size/color capsules (Table 3). Capsule 1 is a brown AA capsule and contains either the hydrocodone/acetaminophen for OPIOID participants or ibuprofen for OPIOID participants. Capsule 2 is a white 00 capsule containing either a placebo for OPIOID or acetaminophen for NON-OPIOID. Capsule 1 for OPIOID and NON-OPIOID is manufactured with a similar weight to the extent possible. Similarly, capsule 2 of OPIOID and NON-OPIOID is manufactured with a similar weight to the extent possible. The study instructions for taking the analgesic are the same for both the opioid and non-opioid cohorts, ensuring the patient, surgeon, and site nurse coordinator are blinded.

**Table 3:** Intervention concealment

Capsule number	OPIOID content	NON-OPIOID content	Quantity for a dose	Total dispensed	Capsule size	Color
1	Hydrocodone 5 mg/acetaminophen 300 mg	Ibuprofen 400 mg	1	20	AA	Brown
2	Placebo	Acetaminophen 500 mg	1	20	00	White

### Implementation {16c}

Prior to the start of the trial, a sequential listing of participant identifiers (IDs) was developed for each gender for each site, resulting in ten separate lists being generated. Subject packages are assembled at the Rutgers CPCC with study analgesic placed into electronic bottles. The electronic bottles contain labels with the subject ID and bottle ID; however, there is no marking on the bottle regarding subject group assignment. Research coordinators at each site distribute subject kits sequentially and are unaware of group assignment.

## Assignment of interventions: blinding

### Who will be blinded {17a}

This is a double-blinded trial. Patients and all clinical site study personnel, including all personnel who interact with subjects, are blinded. Personnel who prepare subject material kits, data management personnel, and project statisticians are unblinded as they have access to group assignment.

### Procedure for unblinding if needed {17b}

Unblinding is only to occur in the case of a medical emergency. If a medical emergency arises, clinical site personnel contact the Clinical Protocol Coordinating Chief who can perform the unblinding by looking up the subjects’ group assignment in the REDCap group assignment project, which is only available to the DMSA and the CPCC staff.

## Data collection and management

### Plans for assessment and collection of outcomes {18a}

All data for this study is being electronically collected via REDCap forms and surveys, electronic medication bottles, and activity monitors.

REDCap forms are being administered by study personnel using an iPad pro tablet. Data collected include eligibility criteria, demographics, and pre-operative patient-reported data (i.e., pre-operative pain, pre-operative sleep ability, and pre-operative pain interference), surgical procedure data (i.e., Surgical Treatment Case Report, including teeth extraction, type of impaction, extraction difficulty, length of surgery, pharmaceuticals used), and post-operative patient-reported data (i.e., post-operative pain, post-operative sleep ability, post-operative pain interference, complications, overall satisfaction). All data collection instruments have been approved by the Rutgers University Institutional Review Board (IRB) and can be found in the electronic essential document binder for the OARS study. These forms are available upon request to the corresponding author.

REDCap surveys are used to capture subject eDiary entries. For these entries, a text message or e-mail is sent to a participant’s cell phone in the morning and evening during the post-operative period with the link to the REDCap surveys. Participants click on the link to access the surveys and make their eDiary entries directly through their cell phones.

SMRxT electronic medication bottles by SMRxT Inc. record the date and time each capsule is removed from the bottle. This provides the number of capsules removed each day and ultimately the total number of capsules removed during the study period, enabling the determination of the number of capsules available for diversion at the end of the post-operative period.

ActiGraph wGT3X-BT by ActiGraph, LLC sleep/activity monitors record active calories, total sleep minutes, and deep sleep minutes.

### Plans to promote participant retention and complete follow-up {18b}

To minimize loss of study participants and/or incomplete data collection, the following activities take place:

- An electronic message is sent to the participant each morning and each evening with a link to the eDiary and a reminder to complete their eDiary entry.
- If the eDiary entry is not made within 2 h, an automatic text message reminder is sent up to two times.
- Research coordinators contact participants by phone to reinforce adherence to study protocol.

Participants receive a $125 payment card (credit, debit, or gift card(s)) at the end of their post-operative visit for participating if they report for their post-operative visit, return all study materials (medication bottle with remaining tablets and activity tracker), and complete the pre-operative survey and the post-operative survey.

If a subject does not report for their post-operative visit, the research coordinators reach out to the subject in an attempt to complete the post-operative survey.

### Data management {19}

#### REDCap

This study makes use of an instance of the REDCap (REDCap version 11.1.0 by Vanderbilt University) application operated and maintained by the Rutgers School of Dental Medicine, Office of Information Technology. This instance is directly integrated with Rutgers University’s Identity Management system, responsible for the enforcing of password complexity requirements and provides a centralized mechanism for the revocation of user access to integrated systems.

Users’ rights within REDCap are only assigned to REDCap projects, instruments, and records that are required for their role within the study. Further restrictions are put in place to ensure that study staff at each of the five sites are granted access to data collected only within that site.

REDCap provides built-in audit trail functionality which logs all user activity including, but not limited to, page views, exporting data, entering data, and viewing or modifying fields.

#### SMRxT

Data collected via the SMRxT electronic pill bottles is stored on the SMRxT Nomi platform, transferred via cellular technology from the SMRxT bottle to Nomi. Nomi is directly integrated with REDCap enabling the download of SMRxT data.

#### ActiGraph

As part of the subject kit return to the CPCC process, the ActiGraph data is downloaded. Any issues with the download are identified. If the file does not successfully download, the ActiGraph is taken out of circulation and returned to the company for trouble shooting and data recovery.

#### Barcoding

Barcodes are used extensively throughout the study to label patient materials. Barcodes are generated at the Rutgers Core site using BarTender by Seagull Scientific. Patient materials are then scanned into REDCap using a barcode scanner attached to dedicated study workstations to reduce human error when entering unique identifiers such as serial numbers or patient IDs into those systems.

Prior to enrolling the first participant in the study, a disaster drill requiring system recover from a backup was successfully completed.

#### Data validation

Whenever possible, structured responses and validation rules have been programmed requiring study participants and research staff to enter valid responses only (i.e., check a yes or no box, select from a list of possible answers such as a Likert scale, date has the format of a date—mm/dd/yy and falls within a certain range). Branching logic in REDCap is used when respondents must provide answer to follow-up questions.

#### Stopping logic

The REDCap system has also been designed to not allow research personnel to proceed with enrolling participants unless the consent process has been completed properly, inclusion criteria have been met, and no exclusion criteria are present by blocking the research coordinator from entering the participant’s mobile phone number or e-mail address. This stopping logic is documented within the REDCap forms.

#### Calculation aids

In order to assist the research coordinators in determining whether visits are occurring in the allowable windows, the electronic data capture (EDC) system has been programmed to calculate the number of days between visit 0 and visit 1 and between visit 1 and visit 2. The EDC system has also been programmed to calculate the age of the participant, allowing the research coordinator to determine if the participant is at least 18 years old.

### Confidentiality {27}

All identifying information (name, address, telephone numbers, date of birth) is stored in REDCap. Only authorized study staff who have the need to see this information have privileges to access the REDCap project. When staff leave the study, access privileges are removed. Each quarter, a review is done to verify that only active study staff have access privileges.

#### Deletion of identifying information

As per Rutgers University policy, once the trial is completed, all identifying information will only be stored for 6 years. After, identifying information will be deleted.

#### Database for public release

Only de-identified information will be released to the public upon request to the corresponding author.

### Plans for collection, laboratory evaluation, and storage of biological specimens for genetic or molecular analysis in this trial/future use {33}

A CLIA waived pregnancy test is performed as part of eligibility determination. There is no plan for collection, laboratory evaluation, and storage of any other biological specimens as part of this protocol.

## Statistical methods

### Statistical methods for primary and secondary outcomes {20a}

All statistical analyses will be performed on an intent-to-treat basis. To account for the repeated measures design and the correlations within the study site, a generalized linear mixed model (GLMM) analysis [82], including mixed model and random effects logistic regression analyses, will be used to analyze the primary and secondary outcomes. In these analyses, participants and sites will be treated as random effects, where appropriate. For each test of an outcome, we define the statistical significance by *p* < 0.05. Bonferroni correction will be applied for multiple testing, where appropriate.

#### Aim 1—Pain experience and patient satisfaction

To test hypothesis 1a, a mixed model analysis will be used to model the pain intensity scores as a function of analgesic groups (non-opioid vs. opioid), day (day of treatment, day 1 and day 2 post-surgery, and last day of follow-up), and day × analgesic group interactions as fixed effects. Participants and sites will be adjusted using nested random effects to account for the possibly stronger within-patient than within-site correlations in the data. Variables such as, but not limited to, age, gender, and treatment variation (e.g., type of anesthesia, administration of a post-surgical steroid, difficulty of surgery, time to complete surgery, etc.) will be controlled as covariates in the statistical model. Numbers of analgesic pills and duration between the first and last doses taken during the day will also be controlled as time-dependent covariates in the statistical model as sensitivity analysis. Mean differences in pain intensity scores between NON-OPIOID and OPIOID groups (*μ*_NonOpioid,day_ − *μ*_Opioid,day_) on the day of treatment, averages from the day of treatment to each of the next 2 days, and the last day of follow-up will be compared using linear contrasts. To test the non-inferiority of the non-opioid analgesics H_0_: (*μ*_NonOpioid,day_ − *μ*_Opioid,day_) ≥ *d* vs. H_1_: (*μ*_NonOpioid,day_ − *μ*_Opioid,day_) < *d*, we chose a small and clinically non-significant difference *d* = 1.0 as the non-inferiority margin [79, 80, 83, 84]. The non-inferiority will be assessed using the one-sided 99.375% [85] or two-sided 98.75% (after Bonferroni correction for 4 tests of pain experience) confidence interval (CI) of *μ*_NonOpioid,day_ − *μ*_Opioid,day_. If this CI completely lies below *d*, we then conclude the non-inferiority of the non-opioid analgesics; if it completely lies below 0, we then conclude the (statistical) superiority of the non-opioid analgesics at the 1.25% level (two-sided) [85, 86]. Summary statistics of numbers of pills per day that participants have taken during the entire period from the day of treatment until post-op will also be reported.

As a secondary outcome, percent of participants removed from the study due to the need for a rescue medication will be compared between NON-OPIOID and OPIOID groups using the random effects logistic regression analysis, with the study site as the random effect.

To test hypothesis 1b, we will calculate the proportion of each satisfaction category (e.g., extremely satisfied, satisfied, dissatisfied, and extremely dissatisfied) and apply the chi-square test to compare between NONOPIOID vs. OPIOID. Generalized logistic regression analysis will be used to compare the distribution of satisfaction between NON-OPIOID vs. OPIOID, controlling for covariates. Patient satisfaction is a one-time measurement (asked on the day of post-op visit). Only the study site will be adjusted as a random effect in the generalized logistic regression analysis.

#### Aim 2—Adverse effects, daily function and sleep quality, and opioid-seeking behavior

##### Adverse events

The rate of the occurrence of adverse events will be compared using the random effects Poisson model with the total number of adverse event occurrences from each patient as the dependent variable and the analgesic group (NON-OPIOID vs. OPIOID) as the independent variable (fixed effects). If patient follow-up time varies, we will include the person days as an offset term. Covariates such as, but not limited to, age, gender, and treatment variation, etc., will be controlled in the statistical models as fixed effects. The same analyses will be repeated to compare the rate for each adverse event.

Percent of participants having to leave the study due to adverse events will also be compared using random effects logistic regression analysis as a secondary outcome. Site will be adjusted as a random effect in the statistical models.

##### Daily function and sleep quality

Mean normal-daily-function and sleep quality ratings will be compared between NON-OPIOID vs. OPIOID using mixed model analysis.

##### Future opioid-seeking behavior

We hypothesize that participants receiving opioid prescriptions to help manage acute pain are more likely to receive at least one additional opioid prescription within 6 months. This outcome will be measured through a PDMP check at the 6-month point. Summary statistics and random effects logistic regression analysis will be used to compare the percent of participants filling opioid prescriptions (yes/no) within 6 months post-surgery between OPIOID and NON-OPIOID groups.

##### Drug diversion

We anticipate that participants receiving 5 days of opioid-containing analgesics will have capsules remaining after their acute pain episode has been resolved. Descriptive statistics will be calculated and percent of participants who have capsules remaining and the number of capsules remaining will be reported.

#### Aim 3—Clinical protocol/decision support tool

To address aim 3, we will develop an optimal [87–89] rule (model), as a function of patient characteristics, to recommend which analgesic to prescribe to maximize a patient’s overall satisfaction. Specifically, our sample will be divided into a training set and a testing set at 1:1 ratio. We will develop the optimal rule [88, 89] in the training set and validate it in the testing set, using the methods of Xu et al. [87].

### Interim analyses {21b}

Other than reviewing and summarizing adverse and serious adverse events, there are no plans for interim analyses.

### Methods for additional analyses (e.g., subgroup analyses) {20b}

#### Heterogeneity and subgroup analyses

To address the possible heterogeneity in pain management and side events in men and women due to sex differences and differences in pain tolerance, we will perform subgroup analysis in men and women separately to ensure the hypothesized differences between the analgesic groups still remain. All the statistical analyses described above will be repeated for men and women respectively as subgroup analyses.

#### Sensitivity analyses—site effect

To assess if the differences in NON-OPIOID and OPIOID vary by study site, we will perform sensitivity analysis by either adding interaction terms with site (i.e., site × analgesic groups, site × day, and site × day × analgesic groups) to the statistical models or using stratified analysis (stratified by site).

### Methods in analysis to handle protocol non-adherence and any statistical methods to handle missing data {20c}

To assess the impact of missing data when we compare the outcomes between OPIOID and NON-OPIOID, sensitivity analyses assuming not missing-at-random (nMAR) and/or a mix of nMAR and missing-at-random (MAR) will be performed using multiple imputation procedures [90–92] to provide a spectrum of possible treatment effect between OPIOID and NON-OPIOID, helpful for describing plausible treatment effects, in the presence of missing data. We will also explore methods to model the missingness mechanism and apply the methods of selection models [93] or use the pattern-mixture models such as the control-based pattern imputation approach, or the tipping-point approach to handle missing data [81, 94–96].

### Plans to give access to the full protocol, participant-level data, and statistical code {31c}

The full protocol and de-identified participant-level data will be available to the public via a written request to the principal investigator.

## Oversight and monitoring

### Composition of the coordinating center and trial steering committee {5d}

**Fig. 3:**
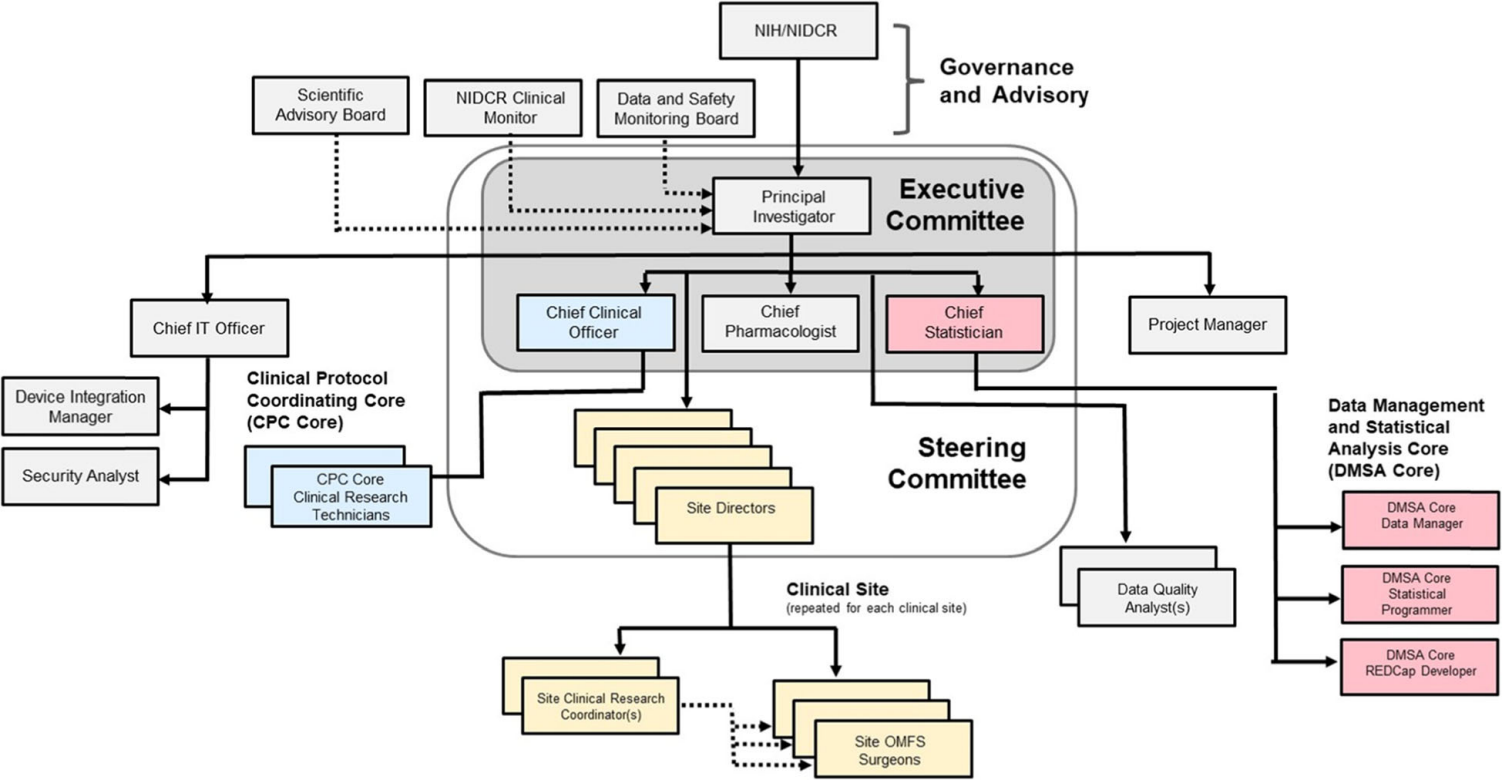
OARS organization chart

Figure 3 contains the organizational chart for the OARS study. There are two coordinating centers. The first, appearing in blue, is the CPCC. This core is responsible for maintaining the protocol, manual of procedures, investigational product (IP), and all materials associated with the trial. In addition, the core is responsible for receiving subject kit orders from the clinical sites, processing the orders which includes assembling the subject kits, receiving all used subject kits from clinical sites, and destroying all materials including leftover investigational product.

The DMSA, which appears in red, is responsible for REDCap project development and upgrades; developing and regular reporting of trial quality metrics including enrolment and retention; randomization of subjects into the OPIOID or NON-OPIOID group; preparing quality management reports for the study team, the Data Safety Monitoring Board (DSMB), the FDA, and the sponsor; locking the database at the end of the trial; and completion of all statistical analyses.

There are two committees overseeing trial implementation. The first, the Executive Committee, is made up of the principal investigator (PI) and the 3 chiefs—the CPCC chief, the DMSC chief, and the chief pharmacologist. The Executive Committee is responsible for overall project management and meets in conjunction with the Steering Committee. Should an emergency meeting be required due to a halting rule being reached or a serious adverse even occurring likely due to IP, the Steering Committee would review the vents to determine the appropriate action. In addition, the Steering Committee is responsible for approving all plans for manuscript submissions.

The Steering Committee consists of all Executive Committee members along with the site directors. The Steering Committee is responsible for the implementation of the protocol at each of the clinical sites. The Steering Committee meets monthly to review the study’s quality metrics, review any adverse events, serious adverse events, protocol deviations, and unanticipated problems. In addition, any changes to the protocol, manual of procedures, clinical quality management plan, or data quality plan are discussed. IT staff and research coordinators routinely attend the Steering Committee Meetings.

### Composition of the data monitoring committee, its role, and reporting structure {21a}

There are two data monitoring committees which provide trial oversight in addition to oversight reporting to the FDA.

NIH-NIDCR has appointed a clinical monitor who is responsible for conducting regular audits. The clinical monitor performs both blinded and unblinded monitoring visits and is responsible for ensuring the protection of human subjects and the integrity of the research data, along with adherence to the study protocol. Blinded monitoring visits focus on the informed consent process and consent records, complete study participant records, event reporting, training of blinded study staff, and maintenance of the projects’ essential documents including IRB records, the protocol, and the manual of procedures. Unblinded monitoring visits focus on investigational product, maintenance of blinding, training of unblinded study staff, and delegation of duties for unblinded staff. Reports are generated from each monitoring visit and shared with the sponsor and the DSMB.

The DSMB is responsible for ensuring study participant safety. The frequency of reviews is dependent upon the risk to the subjects. The DSMB consists of 5 members who are biostatistical, clinical decision support, treatment of addictive disorders, clinical dental pharmacology, and oral and maxillofacial surgery experts. The DSMB reviews recruitment and retention and adverse events. The DSMB periodically reviews accumulated study data for participant safety, study conduct, and progress and, if required, makes recommendations concerning the continuation, modification, or termination of the trial.

### Adverse event reporting and harms {22}

As there are known side effects for all analgesics, subjects may experience adverse effects that are expected side effects, and these are not reported as safety events. All unexpected or serious adverse events are reported however. Safety data are collected through a compilation of adverse events (AEs) captured through daily eDiaries, communication between study team and participant, any event that results in an intermediate clinic visit or hospitalization, or fatality. Safety events associated with interim visits, hospitalizations, and fatalities are reviewed each month at the Steering Committee meeting and are summarized as part of the quarterly Data Quality Management and biannual DSMB reports. Adverse events that result in a hospitalization or fatality are contemporaneously reported to the IRB and the subject sponsor and only serious adverse events related to the study product are contemporaneously reported to the FDA.

### Frequency and plans for auditing trial conduct {23}

In addition to the NIH-NIDCR-appointed monitor and DSMB, the OARS study team performs weekly reviews of all subject records completed that week and performs a quarterly quality management review. This review enables deficiencies to be identified promptly with queries sent to the appropriate site personnel to address identified deficiencies.

A quarterly management review is conducted by the PI, CPCC Chief, and DSMB Chief. During these reviews, 10% of completed records are randomly selected and reviewed for deficiencies. All adverse events, serious adverse events, protocol deviations, and unanticipated problems are reviewed along with the outcome of recommendations developed from the previous review. Adherence to the protocol and manual of procedures is checked along with the maintenance of all study essential documents.

### Plans for communicating important protocol amendments to relevant parties (e.g., trial participants, ethical committees) {25}

All protocol amendments are approved by the Rutgers IRB prior to implementation. Changes are communicated to study staff and the sponsor via a numbered memorandum and reviewed at monthly study staff meetings. The current protocol version along with archived versions of the protocol are maintained in the essential documents electronic binder which is available for review by study staff, monitors, and sponsor.

## Dissemination plans {31a}

This study complies with all applicable NIH Data Sharing Policies [97].

### Dissemination at scientific meetings

Presentations at scientific meetings will be delivered to assist in dissemination of results as soon as possible when final results pertaining to the primary variables are available. Meetings at which presentations will be made include, but will not be limited to, the American and International Association of Dental Research (AADR and IADR), the American Dental Association (ADA), and the International Association for the Study of Pain (IASP) and addiction meetings. NIH grant support will be acknowledged during all presentations.

### Publication and authorship policies

Findings will be published in peer-reviewed journals. Journals selected will be indexed in PubMed. ICMJE guidelines will be adopted and followed in determining authorship. Accepted manuscripts will be submitted to PubMed Central as per NIH policy. Final versions of the peer-reviewed manuscripts will be made available to the public, generally within 3 months but no later than 12 months after the official date of publication. NIH grant support will be acknowledged in all publications.

### NIH Public Access Policy

The NIH *Public Access Policy* requires scientists to submit final peer-reviewed journal manuscripts that arise from NIH funds to *PubMed Central* immediately upon acceptance for publication. This ensures that the public has access to the published results of NIH-funded research.

## Discussion

Dentists often prescribe opioids to their patients to manage acute post-surgical pain, with hydrocodone/acetaminophen being the most commonly prescribed opioid combination. With millions of opioid prescriptions written each year and increasing evidence that adolescents taking opioids are at increased risk of addiction as adults, it is vitally important to develop the best evidence for managing acute post-surgical pain.

Of note is the pragmatic nature of this trial (Table 4). First, unlike most pain studies which follow patients for only a limited number of hours post-surgery, our participants are followed for the normal post-operative period of 1 to 2 weeks covering the entire acute pain episode. Second, randomization is performed within each site, stratified by gender to ensure that results apply equally to men and women with subgroup analysis to be performed. Third, other than the first dose which is required enabling patients to “get ahead of the pain,” we propose using medication the way it is prescribed in clinical practice today. Lastly, our primary study outcome is pain experience and satisfaction with managing pain rather than complete pain relief (total elimination of pain after the pain has risen to a critical level).

**Table 4:** Novel pragmatic aspects of this trial

Clinical situation	Intervention	Outcomes (important to providers and patients)
• Examines gender differences • Allows for extraction of any number of 3rd molars during the surgical visit • Can use any type of anesthesia • Can use post-operative steroids	• Uses medication protocols which can be followed with existing over-the-counter and prescription formularies • Follows decreased FDA-recommended doses for acetaminophen • Allows patients to “get ahead of the pain”	• Follows patients over entire post-operative period (10 days ±5 days) • Primary outcome is pain experience rather than pain relief • Assesses ability to sleep and perform daily activities • Tracks adverse effects • Explores left-over medication which can be diverted • Explores future opioid-seeking behavior

Dental providers are uniquely positioned to take a leadership role in mitigating the national opioid crisis by reducing the number of opioids prescribed to manage acute pain following common dental procedures [98]. This clinical trial will provide key information to impact their prescribing habits.

## Trial status

The OARS trial was activated by the National Institutes of Health on December 29, 2020. The first subject was enrolled on January 7, 2021. It is expected recruitment will be completed by December 2023. The current protocol is version 5.0 dated July 30, 2021.

## Abbreviations

AADR: American Association for Dental Research
AE: Adverse event
BPI: Brief Pain Inventory
CPCC: Clinical Protocol Coordinating CORE
CI: Confidence interval
DMSA: Data Management Statistical Analysis Core
DSMB: Data and Safety Management Board
EDC: Electronic data capture
FDA: Food and Drug Administration
GLMM: Generalized linear mixed model
IADR: International Association for Dental Research
IASP: International Association for the Study of Pain
ICMJE: International Committee of Medical Journal Editors
ID: Identifier
IP: Investigational product
IRB: Institutional Review Board
MAR: Missing-at-random
mg: Milligram
MME: Morphine milligram equivalent
NIDCR: National Institute for Dental and Craniofacial Research
NIH: National Institute of Health
nMAR: Not missing-at-random
NON-OPIOID: 400 mg ibuprofen/500 mg acetaminophen
NRS: Numeric Rating Scale
OARS: Opioid Analgesic Reduction Study
OPIOID: 5 mg hydrocodone/300 mg acetaminophen
PDMP: Prescription Database Monitoring Program
PI: Principal investigator
PROMIS Short 6b: Patient-Reported Outcomes Measurement Information System Adult Short Form v1.0 – Pain Interference 6b
PSQ-3: Pain and Sleep Questionnaire 3 Item Index
PTSS: Pain Treatment Satisfaction Scale
SD: Standard deviation
SDMC: Statistical and Data Management Core
SMS: Short message service
SPIRIT: Standard Protocol Items: Recommendations for Interventional Trials
USA: United States of America

## References

[1] Stephenson J (2020). Drug overdose deaths head toward record number in 2020, CDC Warns. JAMA Health Forum.

[2] Centers for Disease Control and Prevention. CDC – Injury Center - Drug Overdose – Drug overdose deaths. https://www.cdc.gov/drugoverdose/deaths/index.html. Accessed 16 Dec 2021. Page was last reviewed on March 1, 2021 by the CDC.

[3] Centers for Disease Control and Prevention. CDC – Injury Center - Opioids – Opioid basics - understanding the epidemic. https://www.cdc.gov/opioids/basics/epidemic.html. Accessed 16 Dec 2021. March 17, 2021 (Last reviewed Date).

[4] Friedman J, Akre S (2021). COVID-19 and the drug overdose crisis: uncovering the deadliest months in the United States, January–July 2020. Am J Public Health..

[5] Centers for Disease Control and Prevention. CDC – Injury Center - Drug Overdose – Drug overdose deaths – Prescription opioids - overview accessed December 16, 2021. https://www.cdc.gov/drugoverdose/deaths/prescription/overview.html

[6] McCauley JL, Leite RS, Melvin CL, Fillingim RB, Brady KT (2016). Dental opioid prescribing practices and risk mitigation strategy implementation: identification of potential targets for provider-level intervention. Subst Abus.

[7] Bicket MC, Long JJ, Pronovost PJ, Alexander GC, Wu CL (2017). Prescription opioid analgesics commonly unused after surgery: a systematic review. JAMA Surg.

[8] Denisco RC, Kenna GA, O’Neil MG, Kulich RJ, Moore PA, Kane WT, Mehta NR, Hersh EV, Katz NP (2011). Prevention of prescription opioid abuse: the role of the dentist. J Am Dent Assoc.

[9] Miech R, Johnston L, O'Malley PM, Keyes KM, Heard K (2015). Prescription opioids in adolescence and future opioid misuse. Pediatrics.

[10] Rudd RA, Seth P, David F, Scholl L (2016). Increases in drug and opioid-involved overdose deaths - United States, 2010-2015. MMWR Morb Mortal Wkly Rep.

[11] Baker JA, Avorn J, Levin R, Bateman BT (2016). Opioid prescribing after surgical extraction of teeth in Medicaid patients, 2000-2010. JAMA.

[12] Rigoni GC (2003). Drug utilization for immediate- and modified release opioids in the U.S. Division of Surveillance, Research & Communication Support, Office of Drug Safety, Food and Drug Administration, Silver Spring: MD.

[13] Levy B, Paulozzi L, Mack KA, Jones CM (2015). Trends in opioid analgesic-prescribing rates by specialty, U.S., 2007-2012. Am J Prev Med.

[14] McCauley JL, Hyer JM, Ramakrishnan VR, Leite R, Melvin CL, Fillingim RB, Frick C, Brady KT (2016). Dental opioid prescribing and multiple opioid prescriptions among dental patients: administrative data from the South Carolina prescription drug monitoring program. J Am Dent Assoc.

[15] Gabay M (2015). Prescription drug monitoring programs. Hosp Pharm.

[16] Manchikanti L, Helm S, Fellows B, Janata JW, Pampati V, Grider JS, Boswell MV (2012). Opioid epidemic in the United States. Pain Physician..

[17] Hyllested M, Jones S, Pedersen JL, Kehlet H (2002). Comparative effect of paracetamol, NSAIDs or their combination in postoperative pain management: a qualitative review. Br J Anaesth.

[18] Barkin RL (2001). Acetaminophen, aspirin, or ibuprofen in combination analgesic products. Am J Ther..

[19] Singla A, Sloan P (2013). Pharmacokinetic evaluation of hydrocodone/acetaminophen for pain management. J Opioid Manag..

[20] Shah A, Hayes CJ, Martin BC (2017). Characteristics of initial prescription episodes and likelihood of long-term opioid use - United States, 2006-2015. MMWR Morb Mortal Wkly Rep.

[21] Deyo RA, Hallvik SE, Hildebran C, Marino M, Dexter E, Irvine JM, O'Kane N, Van Otterloo J, Wright DA, Leichtling G, Millet LM (2017). Association between initial opioid prescribing patterns and subsequent long-term use among opioid-naive patients: a statewide retrospective cohort study. J Gen Intern Med.

[22] Brummett CM, Waljee JF, Goesling J, Moser S, Lin P, Englesbe MJ, Bohnert ASB, Kheterpal S, Nallamothu BK (2017). New persistent opioid use after minor and major surgical procedures in US adults. JAMA Surg..

[23] Hoppe JA, Kim H, Heard K (2015). Association of emergency department opioid initiation with recurrent opioid use. Ann Emerg Med.

[24] Alam A, Gomes T, Zheng H, Mamdani MM, Juurlink DN, Bell CM (2012). Long-term analgesic use after low-risk surgery: a retrospective cohort study. Arch Intern Med..

[25] Barnett ML, Olenksi AR, Jena AB (2017). Opioid prescribing by emergency physicians and risk of long-term use. N Engl J Med..

[26] O’Neil M (2015). The ADA practical guide to substance use disorders and safe prescribing..

[27] Moore PA, Hersh EV (2013). Combining ibuprofen and acetaminophen for acute pain management after third-molar extractions: translating clinical research to dental practice. J Am Dent Assoc..

[28] Au AH, Choi SW, Cheung CW, Leung YY (2015). The efficacy and clinical safety of various analgesic combinations for post-operative pain after third molar surgery: a systematic review and meta-analysis. PLoS One.

[29] Moore PA, Ziegler KM, Lipman RD, Aminoshariae A, Carrasco-Labra A, Mariotti A, AMA Style (2018). Benefits and harms associated with analgesic medications used in the management of acute dental pain: an overview of systematic reviews [published correction appears in J Am Dent Assoc. 2018 Jun;149(6):413] [published correction appears in J Am Dent Assoc. 2020 Mar;151(3):163]. J Am Dent Assoc.

[30] Moore RA, Wiffen PJ, Derry S, Maguire T, Roy YM, Tyrrell L (2015). Non-prescription (OTC) oral analgesics for acute pain - an overview of Cochrane reviews. Cochrane Database Syst Rev..

[31] Bailey E, Worthington HV, van Wijk A, Yates JM, Coulthard P, Afzal Z. Ibuprofen and/or paracetamol (acetaminophen) for pain relief after surgical removal of lower wisdom teeth. Cochrane Database Syst Rev. 2013;(12):CD004624. 10.1002/14651858.CD004624.pub2 PubMed PMID: 24338830, Epub 2013/12/18. 10.1002/14651858.CD004624.pub2 PMC11561150 24338830

[32] Derry CJ, Derry S, Moore RA. Single dose oral ibuprofen plus paracetamol (acetaminophen) for acute postoperative pain. Cochrane Database Syst Rev. 2013;(6):CD010210. 10.1002/14651858.CD010210.pub2 PubMed PMID: 23794268, Epub 2013/06/25. 10.1002/14651858.CD010210.pub2 PMC6485825 23794268

[33] Bailey E, Worthington H, Coulthard P (2014). Ibuprofen and/or paracetamol (acetaminophen) for pain relief after surgical removal of lower wisdom teeth, a Cochrane systematic review. Br Dent J..

[34] Moore RA, Derry S, Aldington D, Wiffen PJ (2015). Single dose oral analgesics for acute postoperative pain in adults - an overview of Cochrane reviews. Cochrane Database Syst Rev.

[35] Gaskell H, Derry S, Moore RA, McQuay HJ. Single dose oral oxycodone and oxycodone plus paracetamol (acetaminophen) for acute postoperative pain in adults. Cochrane Database Syst Rev. 2009;(3):CD002763. 10.1002/14651858.CD002763.pub2 Epub 2009/07/10. PubMed PMID: 19588335; PMCID: PMC4170904. 10.1002/14651858.CD002763.pub2 PMC4170904 19588335

[36] Toms L, Derry S, Moore RA, McQuay HJ. Single dose oral paracetamol (acetaminophen) with codeine for postoperative pain in adults. Cochrane Database Syst Rev. 2009;(1):CD001547. 10.1002/14651858.CD001547.pub2 Epub 2009/01/23. PubMed PMID: 19160199; PMCID: PMC4171965. 10.1002/14651858.CD001547.pub2 PMC4171965 19160199

[37] Hadhimane A, Shankariah M, Neswi KV (2016). Pre-emptive analgesia with ketamine for relief of postoperative pain after surgical removal of impacted mandibular third molars. J Maxillofac Oral Surg.

[38] Janakiram C, Chalmers NI, Fontelo P, Huser V, Lopez Mitnik G, Iafolla TJ, Brow AR, Dye BA (2018). Sex and race or ethnicity disparities in opioid prescriptions for dental diagnoses among patients receiving Medicaid. J Am Dent Assoc..

[39] Bartley EJ, Fillingim RB (2013). Sex differences in pain: a brief review of clinical and experimental findings. Br J Anaesth.

[40] Gear RW, Miaskowski C, Gordon NC, Paul SM, Heller PH, Levine JD (1996). Kappa-opioids produce significantly greater analgesia in women than in men. Nat Med..

[41] Greenspan JD, Craft RM, LeResche L (2007). Studying sex and gender differences in pain and analgesia: a consensus report. Pain.

[42] Chang AK, Bijur PE, Esses D, Barnaby DP, Baer J (2017). Effect of a single dose of oral opioid and nonopioid analgesics on acute extremity pain in the emergency department: a randomized clinical trial. JAMA.

[43] Daniels SE, Goulder MA, Aspley S, Reader S (2011). A randomised, five-parallel-group, placebo-controlled trial comparing the efficacy and tolerability of analgesic combinations including a novel single-tablet combination of ibuprofen/paracetamol for postoperative dental pain. Pain..

[44] McCabe SE, West BT, Boyd CJ (2013). Leftover prescription opioids and nonmedical use among high school seniors: a multi-cohort national study. J Adolesc Health.

[45] Ashrafioun L, Edwards PC, Bohnert AS, Ilgen MA (2014). Nonmedical use of pain medications in dental patients. Am J Drug Alcohol Abuse..

[46] Maughan BC, Hersh EV, Shofer FS, Wanner KJ, Archer E, Carrasco LR, Rhodes KV (2016). Unused opioid analgesics and drug disposal following outpatient dental surgery: a randomized controlled trial. Drug Alcohol Depend..

[47] Sirintawat N, Sawang K, Chaiyasamut T, Wongsirichat N (2017). Pain measurement in oral and maxillofacial surgery. J Dent Anesth Pain Med.

[48] Meechan JG, Seymour RA (1993). The use of third molar surgery in clinical pharmacology. Br J Oral Maxillofac Surg.

[49] Barden J, Edwards JE, McQuay HJ, Andrew MR (2004). Pain and analgesic response after third molar extraction and other postsurgical pain. Pain..

[50] Cooper SA, Desjardins PJ (2010). The value of the dental impaction pain model in drug development. Methods Mol Biol..

[51] Singla NK, Desjardins PJ, Chang PD (2014). A comparison of the clinical and experimental characteristics of four acute surgical pain models: dental extraction, bunionectomy, joint replacement, and soft tissue surgery. Pain..

[52] Fricke JR, Karim R, Jordan D, Rosenthal N (2002). A double-blind, single-dose comparison of the analgesic efficacy of tramadol/acetaminophen combination tablets, hydrocodone/acetaminophen combination tablets, and placebo after oral surgery. Clin Ther..

[53] Niebler G, Dayno J (2016). Effect size comparison of ketorolac nasal spray and commonly prescribed oral combination opioids for pain relief after third molar extraction surgery. Postgrad Med..

[54] Forbes JA, Bowser MW, Calderazzo JP, Foor VM (1981). An evaluation of the analgesic efficacy of three opioid-analgesic combinations in postoperative oral surgery pain. J Oral Surg..

[55] Hewitt DJ, Todd KH, Xiang J, Jordan DM, Rosenthal NR (2007). CAPSS-216 Study Investigators. Tramadol/acetaminophen or hydrocodone/acetaminophen for the treatment of ankle sprain: a randomized, placebo-controlled trial. Ann Emerg Med.

[56] Turturro MA, Paris PM, Larkin GL (1998). Tramadol versus hydrocodone-acetaminophen in acute musculoskeletal pain: a randomized, double-blind clinical trial. Ann Emerg Med..

[57] Fricke J, Halladay SC, Bynum L, Francisco CA (1993). Pain relief after dental impaction surgery using ketorolac, hydrocodone plus acetaminophen, or placebo. Clin Ther..

[58] Forbes JA, Bates JA, Edquist IA, Burchfield WH, Smith FG, Schwartz MK, Kit V, Hyatt J, Bell WE, Beaver WT (1994). Evaluation of two opioid-acetaminophen combinations and placebo in postoperative oral surgery pain. Pharmacotherapy..

[59] Litkowski LJ, Christensen SE, Adamson DN, Van Dyke T, Han SH, Newman KB (2005). Analgesic efficacy and tolerability of oxycodone 5 mg/ibuprofen 400 mg compared with those of oxycodone 5 mg/acetaminophen 325 mg and hydrocodone 7.5 mg/acetaminophen 500 mg in patients with moderate to severe postoperative pain: a randomized, double-blind, placebo-controlled, single-dose, parallel-group study in a dental pain model. Clin Ther.

[60] Kea B, Fu R, Lowe RA, Sun BC (2016). Interpreting the National Hospital Ambulatory Medical Care Survey: United States Emergency Department Opioid Prescribing, 2006-2010. Acad Emerg Med.

[61] Mutlu I, Abubaker AO, Laskin DM (2013). Narcotic prescribing habits and other methods of pain control by oral and maxillofacial surgeons after impacted third molar removal. J Oral Maxillofac Surg..

[62] Weiland BM, Wach AG, Kanar BP (2015). Use of opioid pain relievers following extraction of third molars. Compend Contin Educ Dent..

[63] Moore PA, Nahouraii HS, Zovko JG, Wisniewski SR (2006). Dental therapeutic practice patterns in the U.S. II. Analgesics, corticosteroids, and antibiotics. Gen Dent.

[64] Steinmetz CN, Zheng C, Okunseri E, Szabo A, Okunseri C (2017). Opioid analgesic prescribing practices of dental professionals in the United States. JDR Clin Trans Res.

[65] Menhinick KA, Gutmann JL, Regan JD, Taylor SE, Buschang PH (2004). The efficacy of pain control following nonsurgical root canal treatment using ibuprofen or a combination of ibuprofen and acetaminophen in a randomized, double-blind, placebo-controlled study. Int Endod J.

[66] Okunseri C, Okunseri E, Xiang Q, Thorpe JM, Szabo A (2014). Prescription of opioid and nonopioid analgesics for dental care in emergency departments: findings from the National Hospital Ambulatory Medical Care Survey. J Public Health Dent.

[67] Mehlisch DR, Aspley S, Daniels SE, Bandy DP (2010). Comparison of the analgesic efficacy of concurrent ibuprofen and paracetamol with ibuprofen or paracetamol alone in the management of moderate to severe acute postoperative dental pain in adolescents and adults: a randomized, double-blind, placebo- controlled, parallel-group, single-dose, two-center, modified factorial study. Clin Ther.

[68] Mehlisch DR, Aspley S, Daniels SE, Southerden KA, Christensen KS (2010). A single-tablet fixed-dose combination of racemic ibuprofen/paracetamol in the management of moderate to severe postoperative dental pain in adult and adolescent patients: a multicenter, two-stage, randomized, double-blind, parallel-group, placebo-controlled, factorial study. Clin Ther..

[69] Mitchell A, McCrea P, Inglis K, Porter G (2012). A randomized, controlled trial comparing acetaminophen plus ibuprofen versus acetaminophen plus codeine plus caffeine (Tylenol 3) after outpatient breast surgery. Ann Surg Oncol..

[70] Mitchell A, van Zanten SV, Inglis K, Porter G (2008). A randomized controlled trial comparing acetaminophen plus ibuprofen versus acetaminophen plus codeine plus caffeine after outpatient general surgery. J Am Coll Surg..

[71] Sniezek PJ, Brodland DG, Zitelli JA (2011). A randomized controlled trial comparing acetaminophen, acetaminophen and ibuprofen, and acetaminophen and codeine for postoperative pain relief after Mohs surgery and cutaneous reconstruction. Dermatol Surg..

[72] Best AD, De Silva RK, Thomson WM, Tong DC, Cameron CM, De Silva HL (2017). Efficacy of codeine when added to paracetamol (Acetaminophen) and ibuprofen for relief of postoperative pain after surgical removal of impacted third molars: a double-blinded randomized control trial. J Oral Maxillofac Surg..

[73] Graudins A, Meek R, Parkinson J, Egerton-Warburton D, Meyer A (2016). A randomised controlled trial of paracetamol and ibuprofen with or without codeine or oxycodone as initial analgesia for adults with moderate pain from limb injury. Emerg Med Australas..

[74] Ong CK, Seymour RA, Lirk P, Merry AF (2010). Combining paracetamol (acetaminophen) with nonsteroidal antiinflammatory drugs: a qualitative systematic review of analgesic efficacy for acute postoperative pain. Anesth Analg..

[75] Cook KF, Dunn W, Griffith JW, Morrison MT, Tanquary J, Sabata D, Victorson D, Carey LM, MacDermid JC, Dudgeon BJ, Gershon RC (2013). Pain assessment using the NIH Toolbox. Neurology..

[76] Evans CJ, Trudeau E, Mertzanis P, Marquis P, Peña BM, Wong J, Mayne T (2004). Development and validation of the Pain Treatment Satisfaction Scale (PTSS): a patient satisfaction questionnaire for use in patients with chronic or acute pain. Pain..

[77] Ayearst L, Harsanyi Z, Michalko KJ (2012). The Pain and Sleep Questionnaire three-item index (PSQ-3): a reliable and valid measure of the impact of pain on sleep in chronic nonmalignant pain of various etiologies. Pain Res Manag..

[78] Kean J, Monahan PO, Kroenke K, Wu J, Yu Z, Stump TE, Krebs EE (2016). Comparative responsiveness of the PROMIS pain interference short forms, Brief Pain Inventory, PEG, and SF-36 Bodily Pain Subscale. Med Care..

[79] Todd KH, Funk KG, Funk JP, Bonacci R (1996). Clinical significance of reported changes in pain severity. Ann Emerg Med..

[80] Gallagher EJ, Liebman M, Bijur PE (2001). Prospective validation of clinically important changes in pain severity measured on a visual analog scale. Ann Emerg Med..

[81] Julious SA (2004). Sample sizes for clinical trials with normal data. Stat Med.

[82] Breslow NE, Clayton DG (1993). Approximate inference in generalized linear mixed models. J Am Stat Assoc..

[83] D’Agostino RB, Massaro JM, Sullivan LM (2003). Non-inferiority trials: design concepts and issues -the encounters of academic consultants in statistics. Stat Med..

[84] Hawker GA, Mian S, Kendzerska T, French M (2011). Measures of adult pain: Visual Analog Scale for Pain (VAS Pain), Numeric Rating Scale for Pain (NRS Pain), McGill Pain Questionnaire (MPQ), Short-Form McGill Pain Questionnaire (SF-MPQ), Chronic Pain Grade Scale (CPGS), Short Form-36 Bodily Pain Scale (SF-36 BPS), and Measure of Intermittent and Constant Osteoarthritis Pain (ICOAP). Arthritis Care Res (Hoboken).

[85] Huitfeldt B, Hummel J, European Federation of Statisticians in the Pharmaceutical I (2011). The draft FDA guideline on non-inferiority clinical trials: a critical review from European pharmaceutical industry statisticians. Pharm Stat..

[86] Lewis JA (2001). Switching between superiority and non-inferiority: an introductory note. Br J Clin Pharmacol.

[87] Xu Y, Yu M, Zhao YQ, Li Q, Wang S, Shao J (2015). Ragulaized outcome weighted subgroup identification for differential treatment. Biometrics..

[88] Qian M, Murphy SA (2011). Performance guarantees for individualized treatment rules. Ann Stat.

[89] Zhao Y, Zeng D, Rush AJ, Kosorok MR (2012). Estimating individualized treatment rules using outcome weighted learning. J Am Stat Assoc.

[90] Hedeker D, Mermelstein RJ, Demirtas H (2007). Analysis of binary outcomes with missing data: missing smoking, last observation carried forward, and a little multiple imputation. Addiction..

[91] Siddique J, Harel O, Crespi CM (2012). Addressing missing data mechanism uncertainty using multiple-model multiple imputation: application to a longitudinal clinical trial. Ann Appl Stat.

[92] Siddique J, Harel O, Crespi CM, Hedeker D (2014). Binary variable multiple-model multiple imputation toaddress missing data mechanism uncertainty: application to a smoking cessation trial. Stat Med.

[93] Roderick JAL (1995). Modeling the drop-out mechanism in repeated-measures studies. J Am Stat Assoc..

[94] Little RJ, Wang Y (1996). Pattern-mixture models for multivariate incomplete data with covariates. Biometrics..

[95] Carpenter J, Kenward M (2013). Multiple imputation and its applications.

[96] Van Buuren S (2012). Flexible imputation of missing data.

[97] National Institutes of Health. Final NIH policy for data management and sharing. https://grants.nih.gov/grants/guide/notice-files/NOT-OD-21-013.html. Accessed 17 Sept 2021. October 29, 2020 (Release Date).

[98] Somerman MJ, Volkow ND (2018). The role of the oral health community in addressing the opioid overdose epidemic. J Am Dent Assoc..

